# Supplementary material for: Identification and characterization of tertiary lymphoid structures in brain metastases
Source: Acta Neuropathol Commun. 2025 May 3;13:91. doi: 10.1186/s40478-025-02007-x (PMC12049775; doi:10.1186/s40478-025-02007-x)
Supplement: Supplementary file 1 — Supplementary material 1 [file 40478_2025_2007_MOESM1_ESM.pdf]

Identification and characterization of tertiary lymphoid structures in brain metastases

Sadaf S. Mughal[1,16*], Yvonne Reiss[2,3,17*], Jörg Felsberg[4], Lasse Meyer[1], Jadranka Macas[2,3], Silja Schlue[1,14], Tatjana Starzetz[2], Karl Köhrer[5], Tanja Fehm[6], Volkmar Müller[7], Katrin Lamszus[8], Dirk Schadendorf[9,18], Iris Helfrich[9,10,19], Harriet Wikman[11], Anna Berghoff[12], Benedikt Brors[1,13,15,16**], Karl H. Plate[2,3,17]**, Guido Reifenberger[4,18]**

[1]Division Applied Bioinformatics, German Cancer Research Center (DKFZ), Im Neuenheimer Feld 280, 69120 Heidelberg, Germany.

[2]Institute of Neurology (Edinger-Institute), University Hospital, Goethe University, Heinrich-Hoffmann-Strasse 7, 60590 Frankfurt, Germany.

[3] Frankfurt Cancer Institute (FCI), Paul-Ehrlich-Straße 42-44, 60596 Frankfurt, Germany.

[4]Institute of Neuropathology, Medical Faculty, Heinrich Heine University and University Hospital Düsseldorf, Moorenstrasse 5, Düsseldorf, Germany.

[5]Center for Biological and Medical Research (BMFZ), Genomics and Transcriptomics Laboratory (GTL), Heinrich Heine University, Universitätsstrasse 1, Düsseldorf, Germany.

[6]Department of Gynecology and Obstetrics, Medical Faculty, Heinrich Heine University and University Hospital Düsseldorf, Moorenstrasse 5, Düsseldorf, Germany & Center of integrated Oncology ABCD.

[7]Department of Gynecology, University Medical Center Hamburg-Eppendorf, Martinistrasse 52, 20246 Hamburg, Germany.

[8]Laboratory for Brain Tumor Biology, Department of Neurosurgery, University Medical Center Hamburg-Eppendorf, Martinistrasse 52, 20246 Hamburg, Germany.

[9]Department of Dermatology, University Hospital Essen, University Duisburg-Essen, Hufelandstrasse 50, 45147 Essen, Germany.

[10]Department of Dermatology and Allergy, University Hospital of Munich, Ludwig-Maximilian-University (LMU), Frauenlobstrasse 9-11, 80337 Munich, Germany.

[11]Department of Tumor Biology, University Medical Center Hamburg-Eppendorf, Martinistrasse 52, 20246 Hamburg, Germany.

[12]Department of Internal Medicine 1, Clinical Division of Oncology, Medical University Vienna, Währinger Gürtel 18-20, 1090 Vienna, Austria.

[13]Medical Faculty and Faculty of Biosciences, Heidelberg University, Germany.

[14] Faculty of Biosciences, Heidelberg University, 69120 Heidelberg, Germany.

[15]National Center for Tumor Diseases (NCT), Im Neuenheimer Feld 410, 69120 Heidelberg, Germany.

[16]German Cancer Consortium (DKTK), Core Center Heidelberg, Germany.

[17]German Cancer Consortium (DKTK), Partner site Frankfurt/Mainz and German Cancer Research Center (DKFZ), Heidelberg, Germany.

[18]German Cancer Consortium (DKTK), Partner Site Essen/Düsseldorf and German Cancer Research Center (DKFZ), Heidelberg, Germany.

[19]German Cancer Consortium (DKTK), Partner Site Munich and German Cancer Research Center (DKFZ), Heidelberg, Germany.

* Joint first authors, ** Joint last authors

Corresponding author:

Dr. Sadaf S. Mughal, Division Applied Bioinformatics, German Cancer Research Center (DKFZ), Im Neuenheimer Feld 280, 69120 Heidelberg, Germany, E-mail: s.mughal@dkfz-heidelberg.de

Supplementary Figures

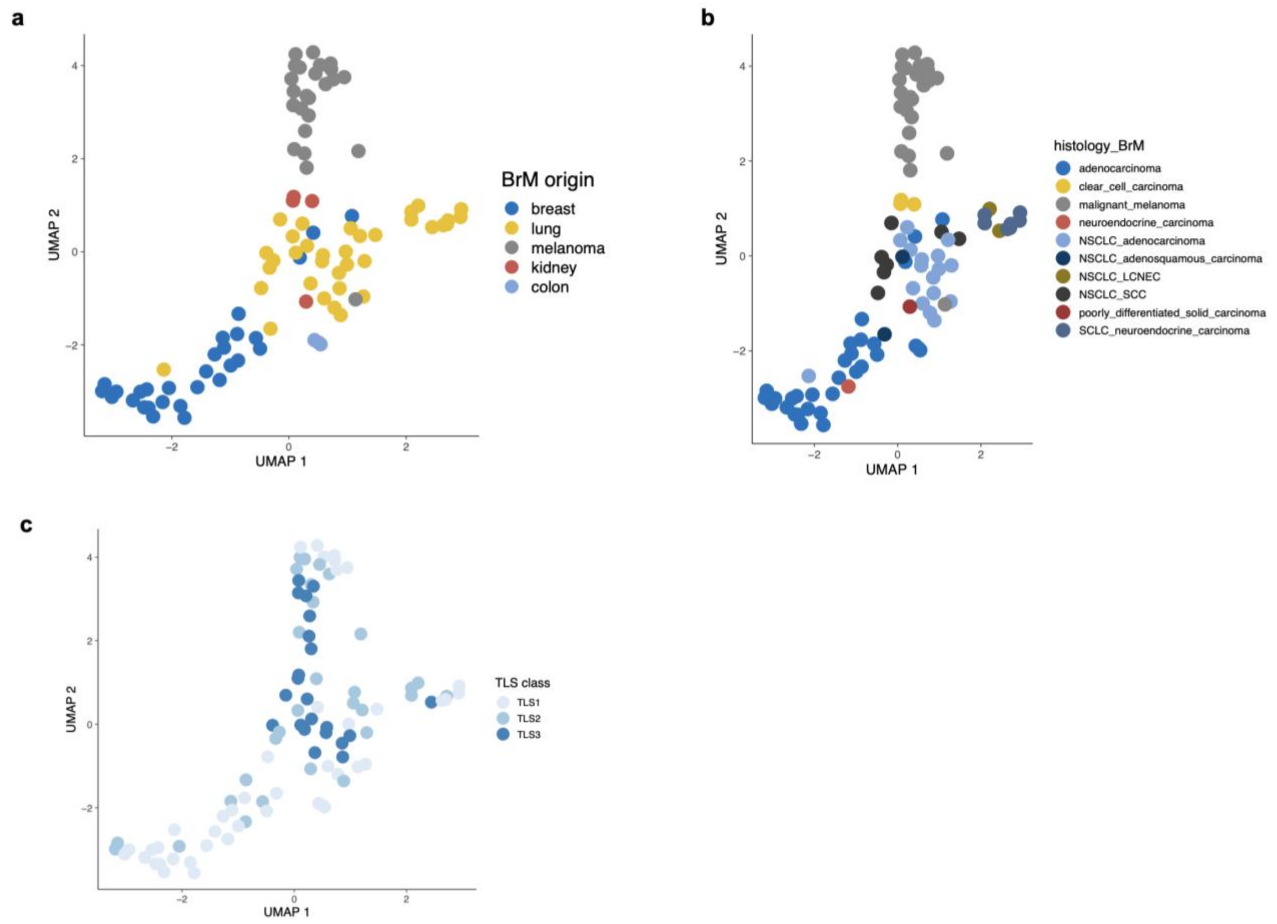

Supplementary Figure S1. Overview of BrM RNAseq cohort. (a) UMAP representation of normalized gene expression data of the BrM cohort according to the primary tumor type of the BrM. **(b)** UMAP representation of normalized gene expression data in the BrM cohort according to the histological classification of the BrM. The lung BrM samples form two clusters and the smaller cluster on the left is mainly composed of BrM from SCLC neuroendocrine tumors and two samples belonging to BrM from NSCLC large cell neuroendocrine carcinoma (LCNEC). **(c)** UMAP representation and assignment of samples to the gene expression-based TLS classes. Color codes for the primary tumors of BrM origin (A), tumor histology (B) and TLS class (C) are provided right to each figure.

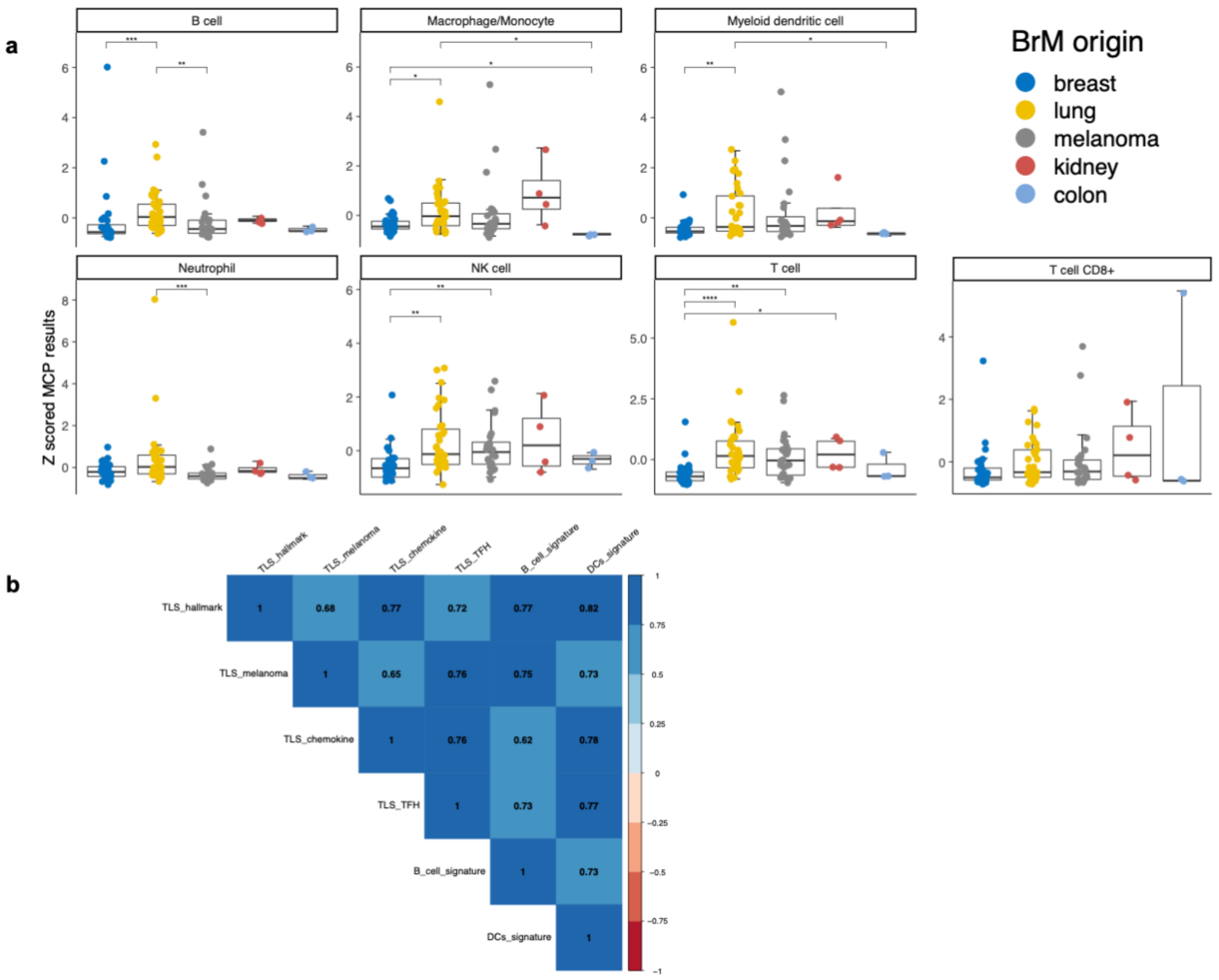

Supplementary Figure S2. Analysis of tumor infiltrating leukocyte compartments according to cell type using MCPcounter [1] **immune cell content inference (a)** Enrichment of cell states in BrM from different primary tumor types in the investigated BrM cohort of breast, lung, kidney and colon carcinomas as well as melanomas. The p-values were corrected for multiple testing using the Benjamini-Hochberg method. **(b)** Spearman correlation based heatmap depicting the associations between TLS gene signatures in BrM.

a

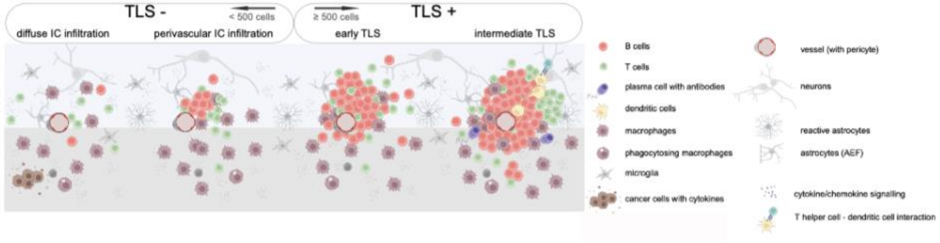

b

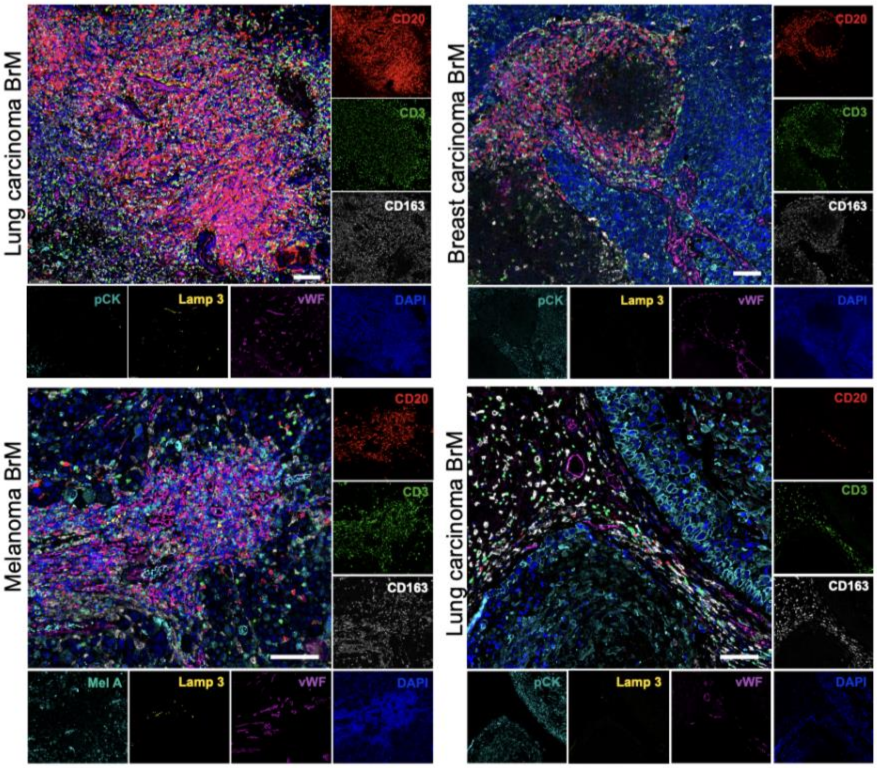

c

BrM Origin	N	TLS high+	TLS intermediate+	TLS low+	No TLS		Number of TLS+ / Tumor Entity
					PV ICs	Diffuse ICs	
Lung	36	4	2	6	16	8	12/36
Breast	14		1	5	2	6	6/14
Melanoma	4			1	3		1/4
Colon	3					3	0/3
Kidney	3			2	1		2/3
	**60**	**4**	**3**	**14**	**22**	**17**	**21/60**
			**21 (35%)**		**39 (65%)**		

d

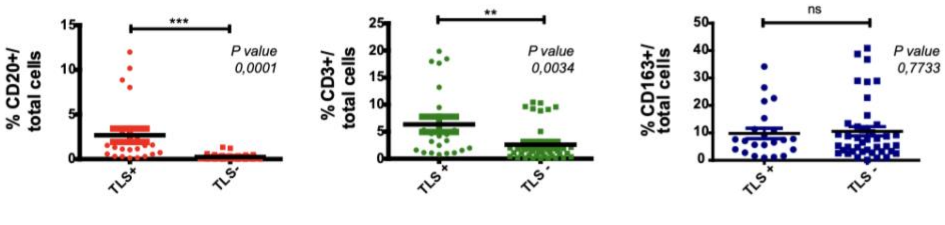

Supplementary Figure S3. Frequency and characteristics of TLS in BrM originating from different primary tumors. (a) Cartoon displaying immune cell infiltration and aggregation pattern of TLS in BrM. TLS were identified as aggregates of CD3+ and CD20+ lymphocytes containing at least 500 cells with a predominance of B cells (>50%; early TLS). In the presence of Lamp3+ dendritic cells, TLS were considered as intermediate TLS. Cases with perivascular immune cell aggregates < 500 cells and diffuse immune cell infiltration were considered as TLS-negative. Cartoon created with BioRender.com. **(b)** Corresponding monoplex images of the exemplary multiplex images shown for the four selected tumors depicted in Figure 3C are displayed. Images captured in a single channel highlight l CD20+ B lymphocytes (red), CD3+ T lymphocytes (green), CD163+ macrophages (white), pCK+ or Mel A+ tumor cells (cyan), Lamp3+ dendritic cells (yellow), vWF+ endothelial cells (magenta) and cell nuclei (blue). Scale bars: 50 µm. **(c)** Further examples of TLS detected in other BrM from lung, breast and kidney cancers are illustrated. **(d)** Table displaying the frequency of TLS in BrM from diverse primary tumors. TLS scores ranged from high to intermediate and low. In TLS-negative cases, small perivascular immune aggregations of T and B cells (<500 cells) and diffuse immune cell infiltration were observed. **(e)** Quantification of CD20+ B lymphocytes, CD3+ T lymphocytes, and CD163+ immunosuppressive macrophages in whole slide multispectral images of BrM (N=60) revealed significantly increased infiltration of B- and T-lymphocytes in TLS+ samples (p=0.001 and p=0.034, respectively) whereas there was no difference in infiltration of immunosuppressive macrophages between TLS+ and TLS- samples (p=0.7733).

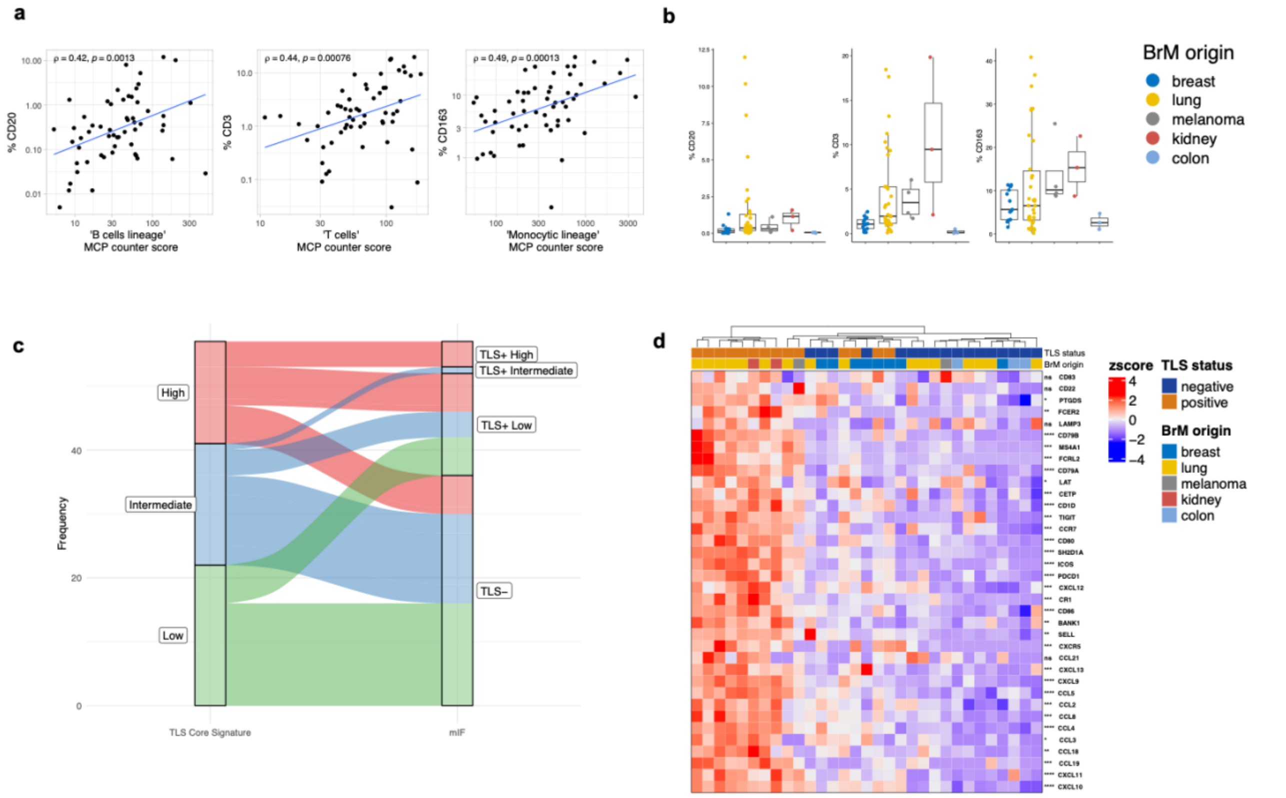

Supplementary Figure S4. Transcriptomic hints for TLS formation in BrM. (a) Correlation of immune cell abundances between the predicted MCP-counter cell scores and multiplex immunofluorescence validations. **(b)** Boxplots displaying the abundances of $CD20^{+}$, $CD3^{+}$ and $CD163^{+}$ cells across BrM from different types by primary cancers. **(c)** River plot showing the overlap of the TLS classification of samples by RNAseq-based TLS signature score and categorization based on multiplex immunofluorescence. **(d)** TLS signature score gene expression in patients with TLS-positive and -negative BrM. The heatmap displays the results of unsupervised hierarchical clustering of the samples (n=31) based on the TLS signature score. Columns and rows are clustered using complete linkage and Euclidean distance.

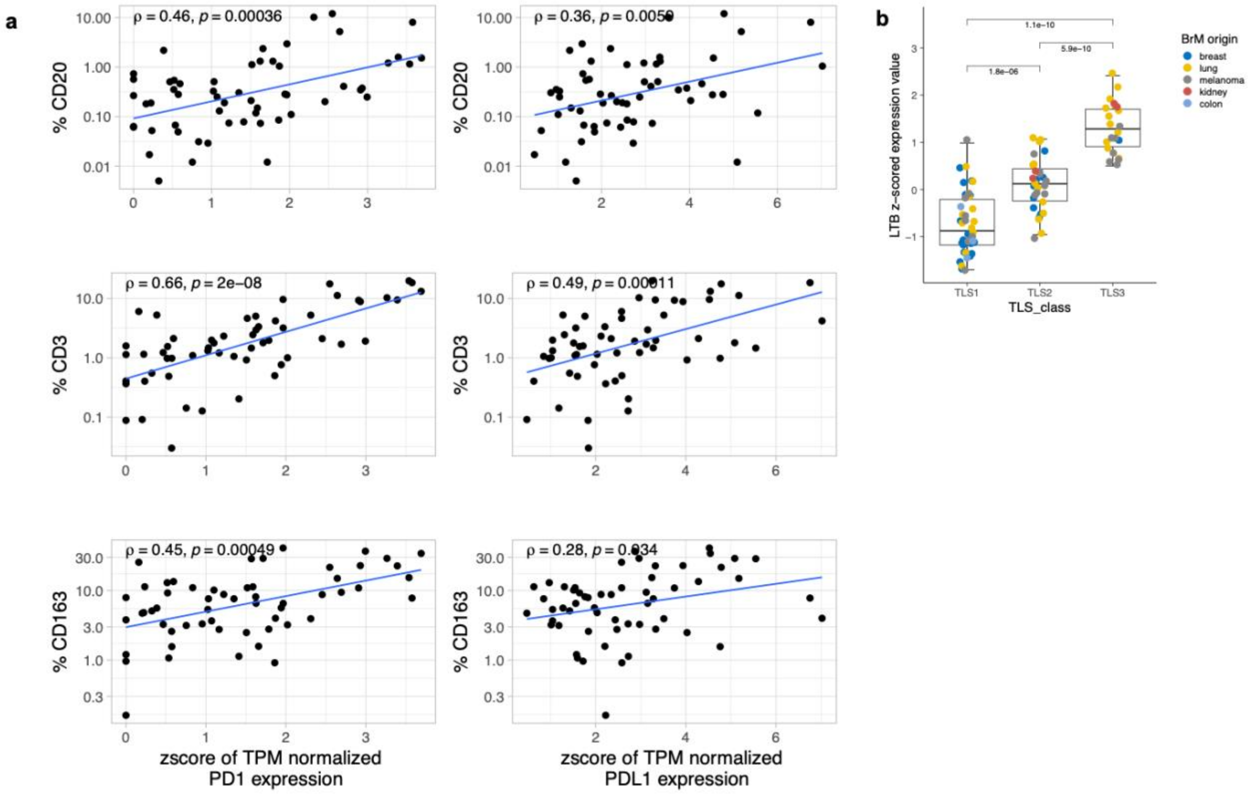

Supplementary Figure S5. Association between lymphoid cell infiltration and expression of major immunomodulatory genes in BrM. (a) Scatter plot displaying the correlation of $CD20^{+}$, $CD3^{+}$ and $CD163^{+}$ cells with PD-L1 and PD-1 gene expression across the investigated BrM samples **(b)** Boxplots displaying normalized gene expression of lymphotoxin beta (LTB) across the TLS classes of BrM. The different BrM origins are highlighted according the color code given right to the graph. Medians are indicated and error bars depict standard deviation. Statistical testing was performed using an unpaired two-sided Wilcoxon test and p-values were corrected for multiple testing using the Benjamini-Hochberg method. Significance levels between groups are: ****, p≤0.0001; ***, p≤0.001; **, p≤0.01; *, p≤0.05; ns (not significant), p>0.05.

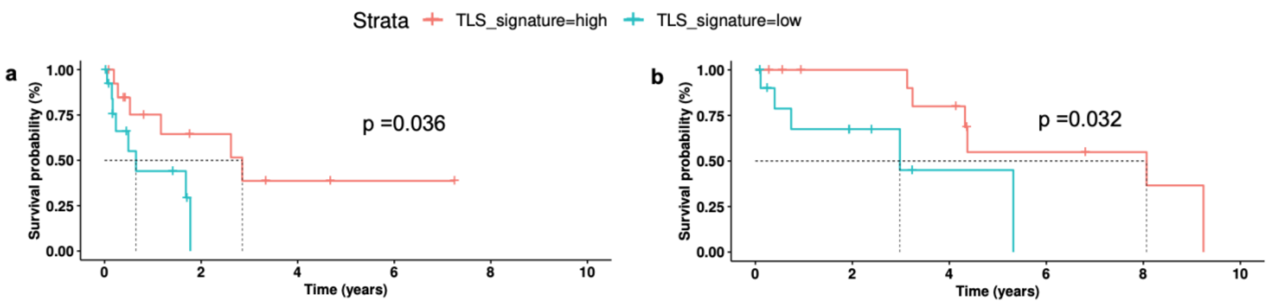

c

Chemotherapy before BrM surgery	TLS high lung BrM patients	TLS low lung BrM patients
Treatment naïve	14	6
Treated	3	11

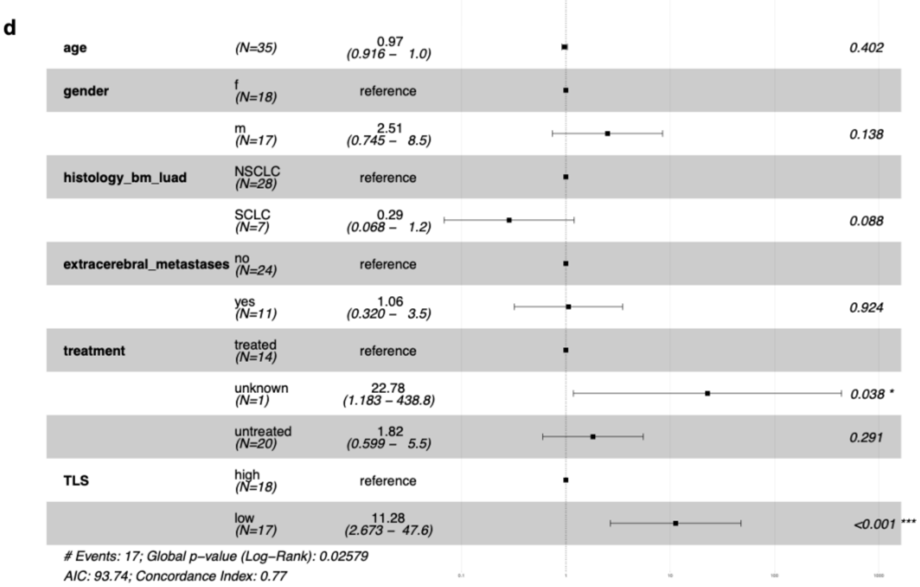

Supplementary Figure S6. Association between TLS class assignment and previous treatment by chemotherapy in patients with BrM from lung cancer and survival in patients with NSCLC BrM. Prolonged survival of patients with BrM from NSCLC with high TLS score in the own institutional cohort **(a)** and **(b)** an independent published cohort [3]**. (c)** In the institutional cohort of 34 patients with BrM from lung cancer, 20 patients had treatment-naïve BrM while 14 patients had received chemotherapy before BrM surgery. Logistic regression using a binominal model showed that TLS high group BrM assignment positively associates with treatment-naïve BrM (p=0.00835). **(d)** Forest plot showing the hazard ratio (HR) and 95% CI of the association of overall survival with different clinical parameters (patient age at diagnosis, patient gender, histological subtype (NSCLS, SCLC), presence of extracerebral metastases, treatment status and TLS high/ low status). Cox proportional

hazard regression model was applied to determine statistical significance. Note that TLS class assignment remained prognostically significant upon multivariate analysis.

Supplementary Tables

Table S1. Clinical characteristics of the BrM patient cohort. The table presents clinical information, such as patient's sex, age at the diagnosis of brain metastasis, localization of the tumor and histological classification.

Table S2. Deconvolution of the BrM RNAseq data. The table presents the results from MCPcounter score values for individual cell types for all patients.

Table S3. TLS signature score. The table presents the expression values (z transformed) for individual genes comprising the TLS signature.

Table S4. Results of mIF analyses from an independent melanoma BrM cohort (n=55) [2]. The table lists B (CD20+) and T (CD3+) lymphocyte frequencies and TLS classification.

Table S5. Differentially expressed genes in TLS-positive and TLS-negative BrM tumors. Only genes with a foldchange value greater than 0.5 and adjusted p values of less than 0.05 are listed here.

References

1. Becht E, Giraldo NA, Lacroix L, Buttard B, Elarouci N, Petitprez F, Selves J, Laurent-Puig P, Sautès-Fridman C, Fridman WH, De Reyniès A (2016) Estimating the population abundance of tissue-infiltrating immune and stromal cell populations using gene expression. Genome Biol 17:218. doi: 10.1186/s13059-016-1070-5

2. Herrera-Rios D, Mughal SS, Teuber-Hanselmann S, Pierscianek D, Sucker A, Jansen P, Schimming T, Klode J, Reifenberger J, Felsberg J, Keyvani K, Brors B, Sure U, Reifenberger G, Schadendorf D, Helfrich I (2020) Macrophages/Microglia Represent the Major Source of Indolamine 2,3-Dioxygenase Expression in Melanoma Metastases of the Brain. Front Immunol 11:120. doi: 10.3389/fimmu.2020.00120

3. Rubio-Perez C, Planas-Rigol E, Trincado JL, Bonfill-Teixidor E, Arias A, Marchese D, Moutinho C, Serna G, Pedrosa L, Iurlaro R, Martínez-Ricarte F, Escudero L, Cordero E, Cicuendez M, Ruiz S, Parra G, Nuciforo P, Gonzalez J, Pineda E, Sahuquillo J, Tabernero J, Heyn H, Seoane J (2021) Immune cell profiling of the cerebrospinal fluid enables the characterization of the brain metastasis microenvironment. Nat Commun 12:1503. doi: 10.1038/s41467-021-21789-x
